# Convergent cross sorting for estimating dynamic coupling

**DOI:** 10.1038/s41598-021-98864-2

**Published:** 2021-10-13

**Authors:** Leo Breston, Eric J. Leonardis, Laleh K. Quinn, Michael Tolston, Janet Wiles, Andrea A. Chiba

**Affiliations:** 1 Program in Neurosciences, University of California, San Diego, La Jolla, CA 92093 USA; 2 Department of Cognitive Science, University of California, San Diego, La Jolla, CA 92093 USA; 3 Air Force Research Laboratory, Wright Patterson Air Force Base, Dayton, OH 4532 USA; 4 School of Information Technology and Electrical Engineering, University of Queensland, Brisbane, QLD 4072 Australia

**Keywords:** Neuroscience, Computational neuroscience, Social neuroscience, Computational biology and bioinformatics, Computational neuroscience, Data mining, Data processing, Software, Statistical methods, Complex networks, Nonlinear phenomena

## Abstract

Natural systems exhibit diverse behavior generated by complex interactions between their constituent parts. To characterize these interactions, we introduce Convergent Cross Sorting (CCS), a novel algorithm based on convergent cross mapping (CCM) for estimating dynamic coupling from time series data. CCS extends CCM by using the relative ranking of distances within state-space reconstructions to improve the prior methods’ performance at identifying the existence, relative strength, and directionality of coupling across a wide range of signal and noise characteristics. In particular, relative to CCM, CCS has a large performance advantage when analyzing very short time series data and data from continuous dynamical systems with synchronous behavior. This advantage allows CCS to better uncover the temporal and directional relationships within systems that undergo frequent and short-lived switches in dynamics, such as neural systems. In this paper, we validate CCS on simulated data and demonstrate its applicability to electrophysiological recordings from interacting brain regions.

## Introduction

Determining the causal relationships between the components of a system is a ubiquitous challenge across the sciences. To this end, many methods have been developed to estimate these interactions from their observed time series. Each method’s domain of applicability is determined by its definition of causality, and its assumptions about the underlying system. Convergent Cross Mapping (CCM) is an approach, based on state space reconstruction (SSR) (also referred to as phase space reconstruction), which is best suited for complex, nonlinear systems, such as those found in neuroscience, ecology, and the social sciences^1–9^. These systems’ myriad feedback loops and deterministic components cause the information about their variables to become inseparably mixed^1^. This presents a challenge to methods based on stochastic processes, such as Granger Causality (GC), because they define causation as the ability of one process to provide additional predictive information about another^10^. These assumptions may not be as suitable for deterministic systems because every coupled variable carries information about the others, meaning that variables cannot be fully removed from the system for analysis, which violates the assumptions of GC^1^.

In contrast, CCM tests for causal coupling by measuring the correspondence between the SSRs produced from time series of two different variables. If there is a smooth mapping between them, then both variables are likely part of the same dynamical system and thus deterministically coupled. CCM has been successfully applied to a diverse range of systems, including fisheries, online social networks, and fMRI^1,11,12^.

Despite its success, CCM has several practical problems that have been noted in the literature: It requires a large number of samples to converge, it struggles in cases containing strongly coupled variables or synchrony, and its performance degrades with noise^5,7,8,13^. Subsequent work has tried to address some of these problems. For instance, Ye et al. introduced lagged CCM estimates to improve performance on strongly coupled variables, while Ma et al. introduced Cross Map Smoothness to reduce the required time series length^2,8^. Though these approaches were successful, they only address individual failure modes.

To improve CCM’s performance on issues related to coupling strength, noise, and sample size, we propose a new implementation known as Convergent Cross Sorting (CCS). CCS measures the correspondence between reconstructed manifolds by comparing the relative ranking of the pairwise distances between samples. This approach affords multiple advantages including selectively sampling the most informative distances and normalizing for geometric transformations that distort absolute distance but preserve relative order.

In this paper we validate CCS’s ability to identify the existence, directionality, and relative strength of coupling relationships for a wide range of simulated signal and noise characteristics. We also highlight the importance of using CCS on well-characterized systems with known structural connectivity informed by the functional dynamics reported in prior literature. As an exemplar, we demonstrated the multiple uses of CCS as applied to neural recordings from known anatomical circuits to examine dynamic network states during complex behaviors.

## Results

### Theoretical validation

#### Simulated data

**Figure 1:**
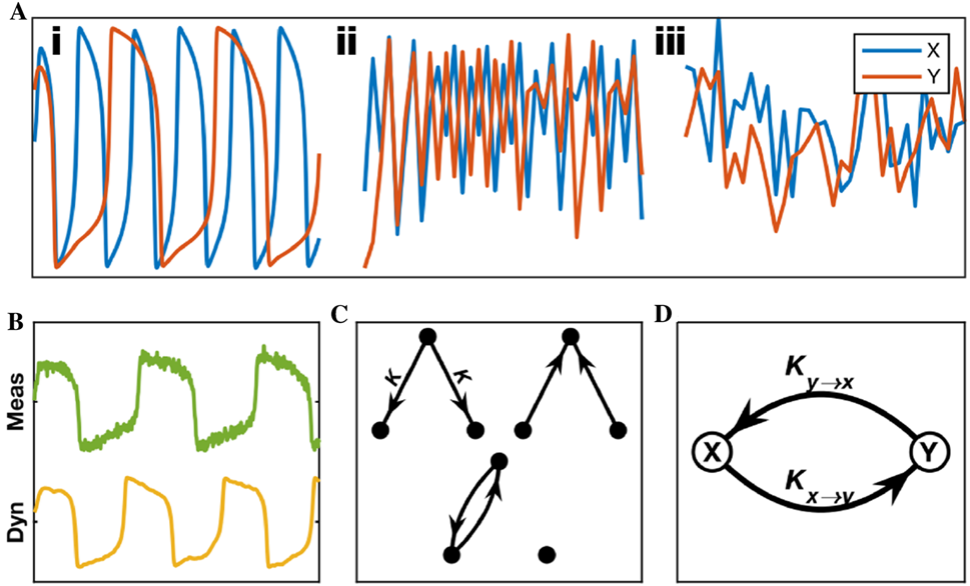
Simulated Data. (**A**) Time series from the three classes of model used for validation: (i) Van der Pol Oscillators, (ii) Logistic Maps, (iii) Autoregressive Models. (**B**) Trace of a VDP with measurement noise (top) and (Bottom) dynamic noise. (**C**) Types of causal networks used to generate trials for assessing detection accuracy. Three variable networks afford the ability to test a method’s performance in the presence of third party confounds such as the common driver of two uncoupled variables in (Top Left). All other three variable topologies had to be omitted because they contain transitive causal relationships which leads to ambiguous pairwise results. In each network the coupling strength, $K$, of both edges is the same. (**D**) Two variable networks used to test the response to coupling parameters. ${K}_{x\to y}$ and ${K}_{y\to x}$ can vary independently.

To validate CCS, we compared its performance to CCM on simulated data sets for which the true coupling parameters were known. To cover a wide range of potential signal properties, we considered three types of model systems: Van der Pol oscillators (VDP), Logistic Maps (LM) and Autoregressive Models (AR) (Fig. 1A, SI Simulated Data). VDPs are deterministic, continuous, and approximately periodic, LMs are deterministic, discrete, and chaotic, and ARs are linear and stochastic. ARs were included to test the SSR methods’ performance when applied to processes which violate their underlying assumption of nonlinearity and have a high degree of dynamic stochasticity.

Additionally, to test the methods’ robustness to noise, we corrupted the signals with both measurement noise, $\varepsilon$, and dynamical noise, $\epsilon$. (Fig. 1B, SI Simulated Data) Measurement noise was simulated by adding gaussian noise to the final output time series, while dynamical noise was injected into the system’s ongoing dynamics. To normalize the units for measurement noise we used the Signal to Noise ratio ($SNR$) of the magnitude of the uncorrupted time series to the magnitude of the added noise.

#### Detection accuracy

**Figure 2:**
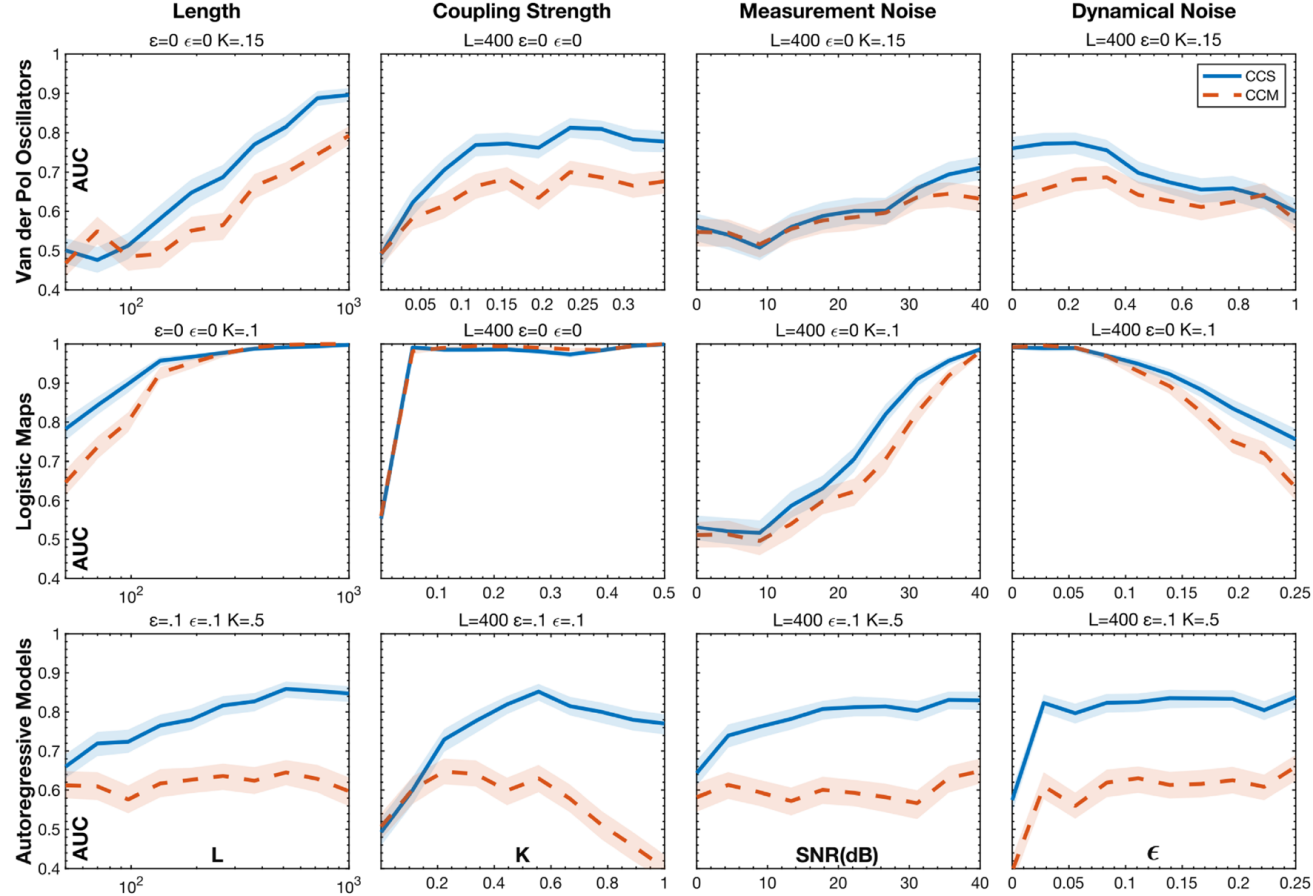
The ROC AUC of CCS and CCM for detecting causal coupling in networks of three variables as a function of signal type, time series length, coupling strength, measurement noise, and dynamical noise. For each condition, the area under the curve (AUC) was calculated using 200 trials of three variable networks. (See Supplementary Area Under the Curve 3 Variable for more information on the accuracy quantification and Table S1 and Choice of Embedding Parameters for detailed methods) The shaded boundaries represent the 95% confidence intervals of the AUCs. For an additional comparison with Granger Causality see Supplementary Fig S1.

Figure 2 compares the accuracy of CCS and CCM at identifying the causal relationships in networks of three variables (Fig. 1C) for varying time series length, $L$, coupling strength, $K$, $SNR$, and $\epsilon$. Each method’s accuracy was quantified using the Area Under the Curve (AUC) of the Receiver Operating Characteristic (ROC). This is a measure of how well an ideal classifier could separate the coupled versus non-coupled time series and corresponds to the probability that a method will score a randomly chosen coupled relationship higher than a non-coupled one.

CCS broadly outperformed CCM on the deterministic systems (VDPs and LMs). It had the largest advantage on VDPs, maintaining an approximately 0.1 higher AUC than CCM for trials with $L>100$ and $K>.04$. CCS also had better accuracy for $\epsilon <.78$, at which point the system became too noisy for either method to perform much above chance. While both methods, were degraded by measurement noise, CCS had slightly better accuracy for $SNR>30 \mathrm{dB}$.

For LMs, CCS and CCM both had excellent accuracy under noise free conditions, however, CCS had much higher accuracy on short trials with $L<100$. At $L=50$, CCS had an AUC of .78 compared to CCM’s .64. CCS also had better accuracy in the case of high dynamical noise with an AUC of .75 at the highest $\epsilon$ value.

#### Relative bidirectional coupling strength

**Figure 3:**
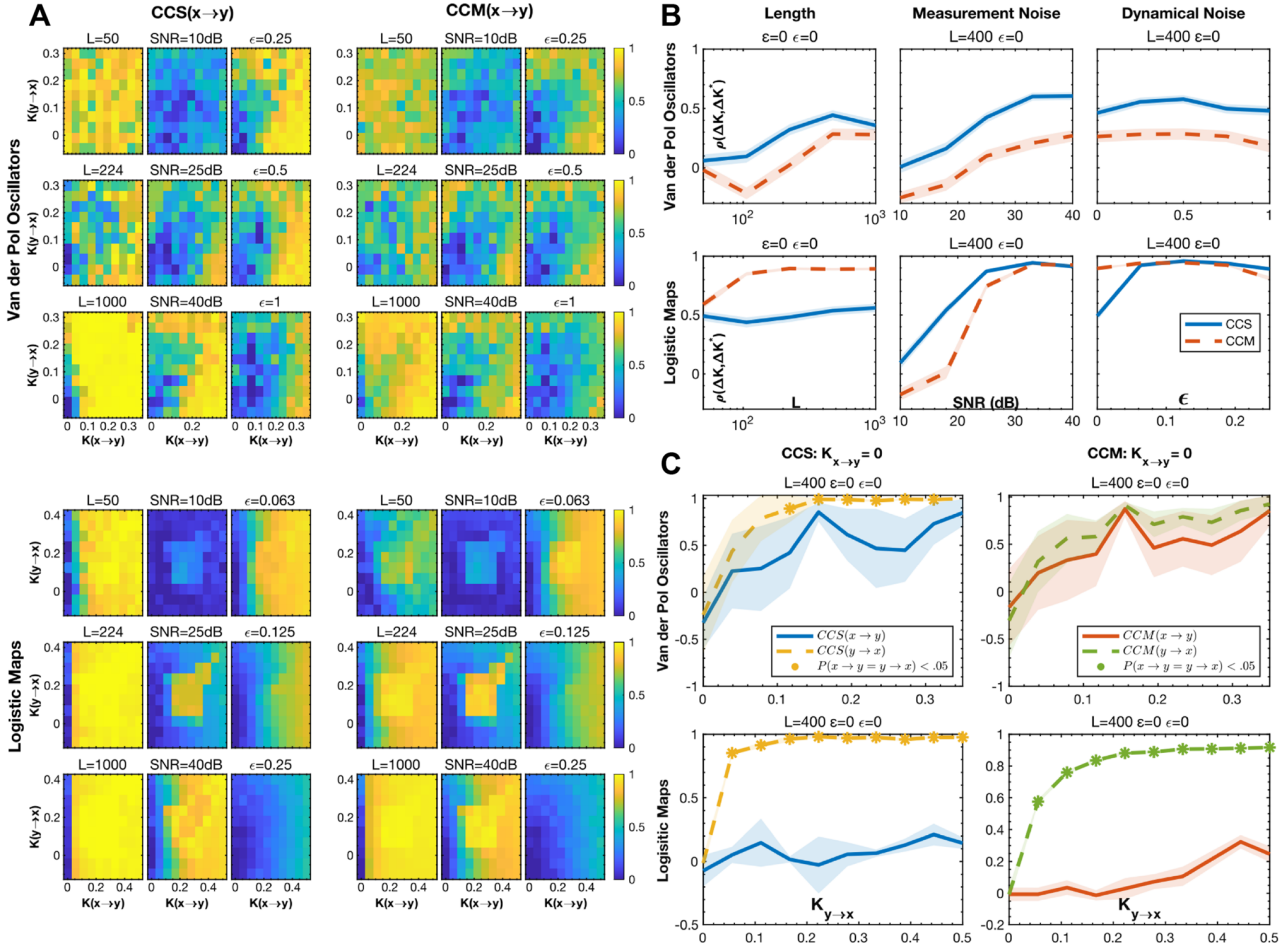
A comparison of CCS and CCM’s ability to determine the relative strength and directionality of coupling. (**A**) CCS and CCM scores for the drive from $x\to y$ as a function of the ${K}_{x\to y}$ and ${K}_{y\to x}$, signal type, $L$, SNR, and $\epsilon$. $L=400$, SNR = $\infty$, and $\epsilon =0$, unless otherwise specified. (**B**) The Spearman correlation between the true difference in coupling strength, ${(K}_{x\to y}-{K}_{y\to x})$ and the estimated one, $score\left(x\to y\right)-score\left(y\to x\right)$. (**C**) CCS and CCM $score\left(x\to y\right)$ and $score(y\to x)$ as a function of ${K}_{y\to x}$ where ${K}_{x\to y}=0$ (i.e., unidirectional coupling). The asterisks show the points at which the means of the two distributions of scores were significantly different. The shaded regions in (**B**) and (**C**) represent 95% confidence intervals.

Beyond identifying the existence of causal coupling, it is also desirable to know the relative magnitude of the interactions between bidirectionally coupled variables, such as those shown in Fig. 1D. This case is widely applicable to many complex natural systems that have ubiquitous feedback loops. Figure 3A shows the CCS and CCM scores for the coupling from $x\to y$ as a function of the generating parameters, ${K}_{x\to y}$ and ${K}_{y\to x}$. Ideal performance would look like a graded increase from left to right and no variation from top to bottom. This would represent a monotonic response to coupling strength without any dependence on the drive in the opposite direction.

To quantify how well these scores reflect the true, relative coupling strength, we found the Spearman correlation between the difference in estimated strength and the difference in generating coefficients, $\rho =corr\left( score\left(x\to y\right)-score\left(y\to x\right),{K}_{x\to y}-{K}_{y\to x}\right)$. Since the differences are signed, $\rho$ captures each method’s accuracy in estimating both the magnitude and direction of the difference in coupling parameters. Furthermore, since the Spearman correlation is a rank statistic, it is strictly testing for the monotonicity of the relationship.

Figure 3B shows the $\rho$ of CCS and CCM as function of system type, $L$, $SNR$, and $\epsilon$. CCS has much better accuracy for every condition other than very low noise LMs. CCM actually has a negative correlation for VDPs with $L<200$ and $SNR<20 \mathrm{dB}$ and LMs with $SNR<20 \mathrm{dB}$, which means that its score systematically misidentified the direction of the coupling. This incorrect bias can be seen clearly in the top right quadrant of Fig. 3A where higher ${K}_{y\to x}$ decreases $score\left(x\to y\right)$ for constant ${K}_{x\to y}$ above moderate values.

The first column in the bottom left quadrant of Fig. 3A shows that CCM outperforms CCS on low noise LMs because CCS saturates at weak coupling strengths. The second two columns demonstrate how small amounts of noise significantly improve the CCS correlation by preventing this saturation.

#### Unidirectional coupling

Figure 3C shows how well the methods differentiated between bidirectional and strong unidirectional coupling. The graphs contain the CCS and CCM scores for $x\to y$ and $y\to x$ as a function of ${K}_{y\to x}$ while holding ${K}_{x\to y}=0$. Both methods performed very well on LMs. They accurately identified the direction of coupling, and their scores for $x\to y$ remained close to zero even at high values of ${K}_{y\to x}$. Each method had small tradeoffs: The CCM estimate for $x\to y$ had a slightly larger dependency on ${K}_{y\to x}$, increasing from $\approx 0$ to $\approx .2$, and the CCS estimate had a higher variance. The VDPs presented a more difficult challenge because they are much more susceptible to synchrony. Both methods’ scores for $x\to y$ had a strong dependency on ${K}_{y\to x}$. However, only the CCS scores were statistically distinguishable. The mean of its score for $y\to x$ was significantly greater than that of $x\to y$ for trials with ${K}_{y\to x}>.1$.

### Applications

#### Neural recordings

**Figure 4:**
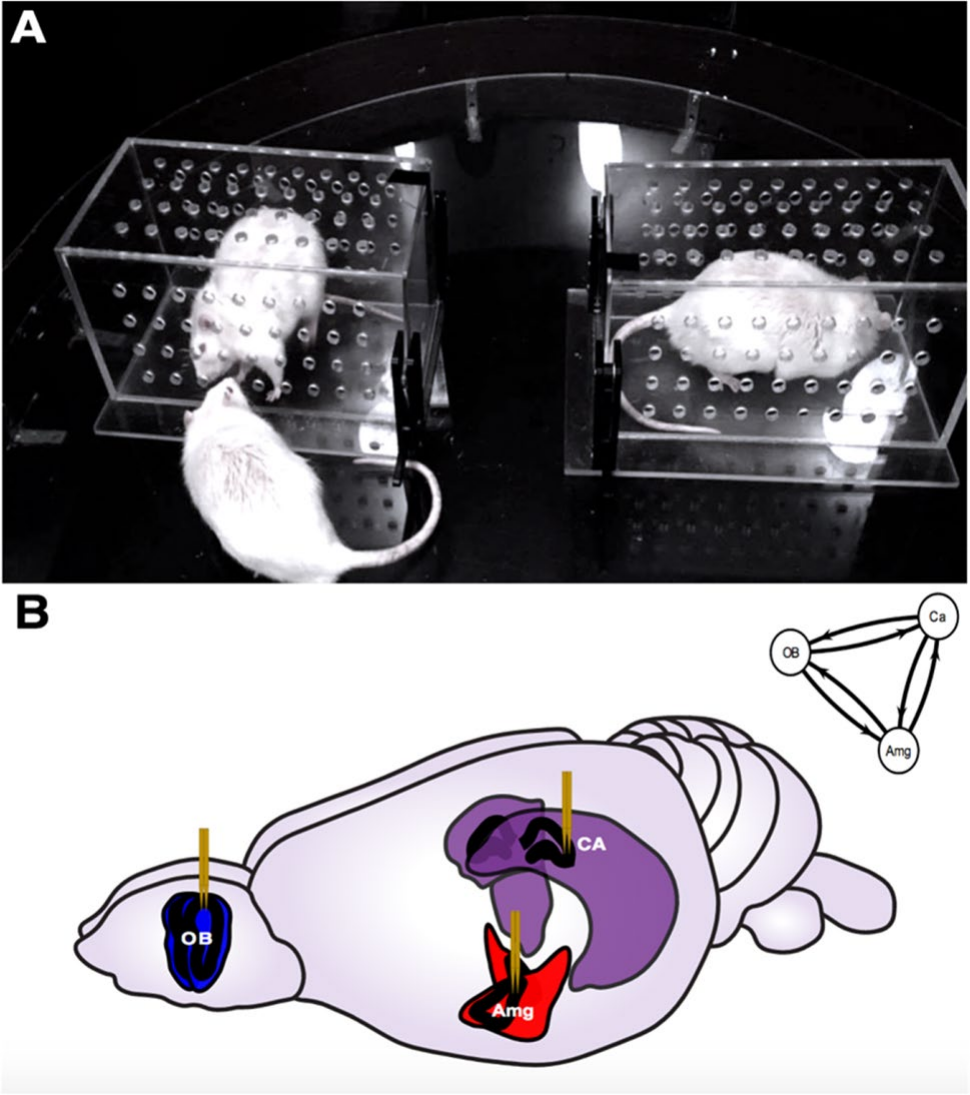
Neural experimental setup. (**A**) Two rats were placed in separate Plexiglas enclosures, while an implanted rat on the outside was free to roam the field and sniff through the holes in those enclosures. The implanted rats were presented with both a novel and a familiar rat. The implanted rat freely roams the field and investigates either the novel or familiar rat. Rats were removed from the field 2 min and 30 s after the onset of a trial. Trials were counterbalanced to control for place preferences, so novel and familiar rats were presented on alternating sides of the field with each trial (See SI for more info on Social Interaction Task and Animals and Housing). (**B**) Rats were surgically implanted with electrodes for electrophysiological recordings in the main olfactory bulb (OB), hippocampus (CA) and medial amygdala (Amg) (See SI for more details on Surgery and Neural Recordings). Figure adapted from scidraw.io under Creative Commons 4.0 license^16^.

To demonstrate the effectiveness of CCS at revealing directional relationships in noisy real-world data, it was applied to estimate dynamic coupling in time series data collected from the olfactory bulb (OB), hippocampus (Ca), and amygdala (Amg) during social interaction and self-grooming behavior of laboratory rats. The social behavior of interest is the olfactory investigation of another conspecific (Fig. 4A). Self-grooming behavior promotes hygiene maintenance and involves tactile self-soothing, as well as olfactory self-investigation (See Behavioral Video Coding in SI). Oscillatory activity was measured by examining local field potentials (LFPs) (Fig. 4B) occurring at different anatomical points in a neural circuit proposed to be important for social memory processing^14^ (See Surgical Procedure and Neural Recordings in SI). This network has previously been shown to elicit increased coupling during social behavior^15^. The application of CCS to this simultaneous multi-region LFP data allows for the examination of the coupling strength and direction of coupling between these reciprocally connected brain structures during complex behavioral changes. These relationships are determined by the system’s anatomical and functional connectivity.

#### Structural connectivity

The amygdala shares reciprocal connectivity with the main olfactory bulb^17^. The amygdala and hippocampus share strong bidirectional connectivity^18^. Amygdalar activity can also play a key role in influencing hippocampal activity through amygdalo-entorhinal networks^19^. The hippocampus and the olfactory bulb also share connectivity in both directions^20^.

#### Functional connectivity

The olfactory bulb LFP contains rich information not only about smell but also the autonomic nervous system, by generating respiratory rhythms that follow inhalation and exhalation cycles^21,22^. Hippocampal theta, one of the most well characterized oscillations in the brain, is associated with spatial mapping and memory^23^. Respiration-coupled activity has also been found in hippocampus^24–26^. The OB and hippocampal LFP exhibit coupling during odor discrimination^20^. The amygdala LFP has been associated with the formation of emotional memories, and exhibits coupling with hippocampus^27^.

#### Average network state

**Figure 5:**
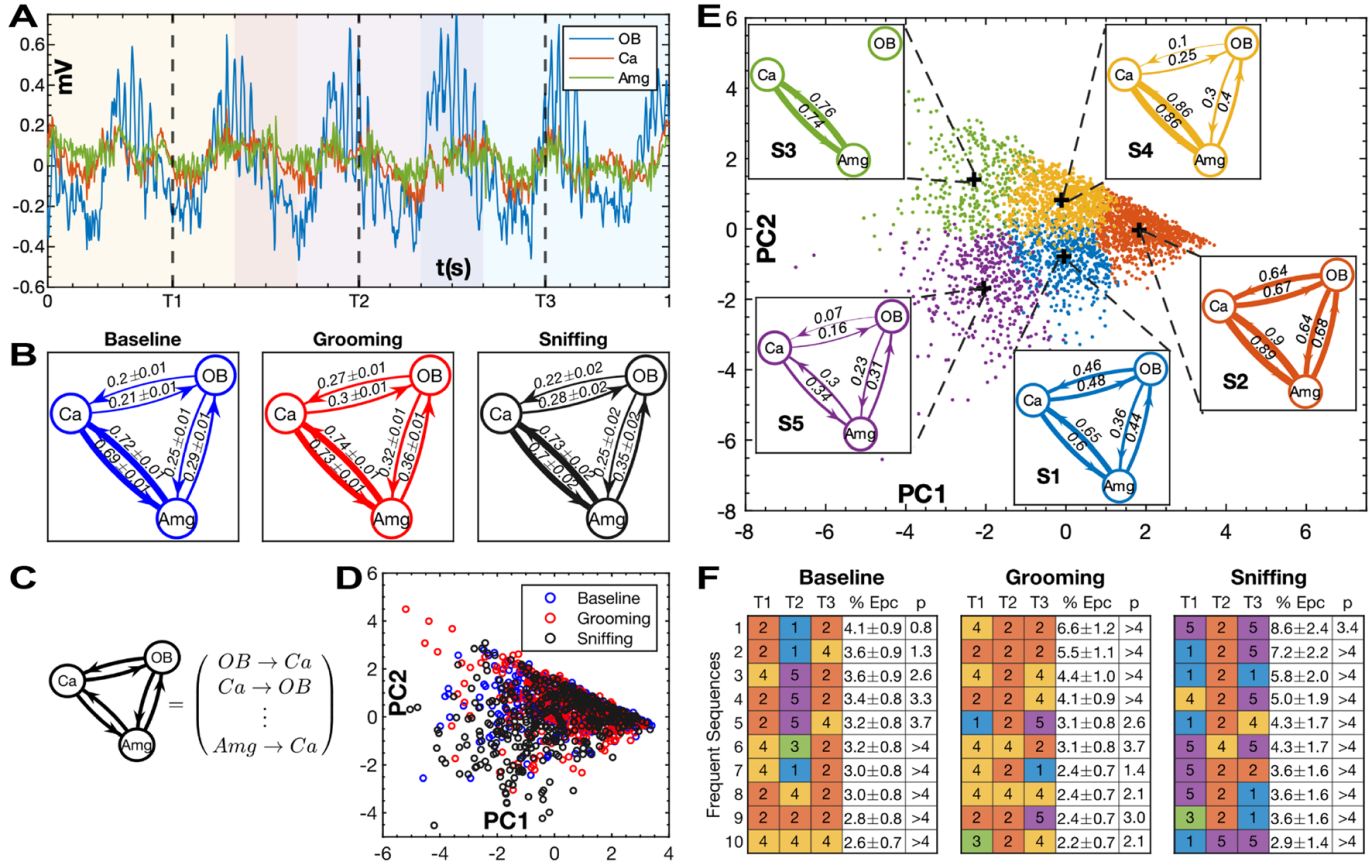
Application of CCS to multi-region neural recordings in rats. (**A**) Example LFP trace from a 1 s epoch. The blue, red, and green lines represent the signals from the Main Olfactory Bulb (OB), Hippocampus (Ca) and Amygdala (Amg), respectively. The time points, T1, T2, and T3 are the centers of 400 ms windows shown by the shaded regions of the plot. (**B**) Average 1 s CCS scores between the three regions during baseline, grooming, and sniffing behavioral epochs (See Supplementary Table S1 and Choice of Embedding Parameters for method details). The error values represent the SEM of each score. All of the scores have a significance $<{10}^{-5}$. (**C**) Illustration of how the CCS scores can be represented by a 6-dimensional vector. (**D**) The distribution of 400 ms CCS scores colored by type of behavior and plotted using the first two principal components. (**E**) The distribution of 400 ms CCS scores colored according to k-means cluster using five means. The inserted graphs show the network diagram corresponding to the centroid of the cluster indicated in the bottom left corner. The values in these diagrams have been corrected for the normalization and whitening transformations used for the PCA. Edges with negative values have been omitted. (**F**) Tables with rows showing the most frequent temporal sequences of CCS states during epochs from each of the behavioral conditions. The first three columns are the moving CCS estimates labeled and colored according to their cluster from (**E**). The 4th column is the percentage of epochs with that sequence. The error value is the standard error of the percentage. The 5th column is the negative log of the probability that the nth most frequent pattern would have a frequency as extreme as the one observed.

Figure 5B shows the average CCS scores for each behavior. The coupling was strongest between CA and Amg for every condition, which is consistent with the regions’ degree of anatomical connectivity. Grooming exhibited the highest overall coupling, containing the maximum score for every edge in the network. Sniffing saw the largest asymmetry in the reciprocal coupling between regions. During sniffing the drive from CA to OB and Amg to OB was 27% and 40% higher, respectively, than the coupling in the reverse direction. In the other two behaviors, the reciprocal coupling differs by no more than 16%.

In general, the results (in Fig. 5B) follow previously established patterns of connectivity. For example, as expected, CCS demonstrated that the hippocampus and amygdala share more connectivity in general than with the distant olfactory bulb^17,18^. These results suggest that the amygdala and olfactory bulb share increased coupling during social investigation relative to baseline, and that the amygdala and hippocampus both show a larger influence over the MOB than in the feedforward direction. During the grooming behavior, all regions showed increased coupling relative to baseline, with more balanced bidirectional coupling between regions than the social sniffing behavior.

#### Temporal dynamics of network states

For higher temporal resolution, we computed 6-dimensional CCS scores using a moving window on each 1 s epoch. (Fig. 5A, C). Figure 5D shows a scatterplot of the first two principal components of these scores, colored according to behavioral type. The plot doesn’t show obvious clustering according to behavior, meaning that at that time scale the different behaviors are composed of varying distributions of similar network states.

To understand the temporal dynamics of the network, we analyzed each epoch’s trajectory through the 6-dimensional coupling space. To make comparisons more tractable, we quantized the space using five k-means clusters which assigned every CCS score to one of five network states (Fig. 5E)^28^.

Figure 5F shows the most common sequences of network states during the epochs from each type of behavior. Since the epochs were extracted from larger events, and from video with a lower sampling rate than the neural data, the phase of the sequences is not informative and cyclic permutations should be considered the same. The three behaviors differed in both the specific highly represented patterns, and the general distribution of sequences. Baseline tended to oscillate from high to moderate coupling, grooming remained in a consistent highly coupled state, and sniffing oscillated between low and high coupling. Baseline also had the flattest distribution of sequences. The top ten most frequent sequences in that condition comprised between 4.1 and 2.6% of the epochs, while the most frequent sequence in grooming and sniffing comprised between 6.6 and to 2.2% and 8.6% to 2.9%, respectively. This result makes sense since baseline is the least restrictive of the behavioral conditions which means it should have the most diverse temporal patterns. Taken together, the results in Fig. 5D and F show that the dynamics of the network during the three behaviors are composed of similar bases of states but differ in their distribution of sequences.

## Methods

### Convergent cross sorting

**Figure 6:**
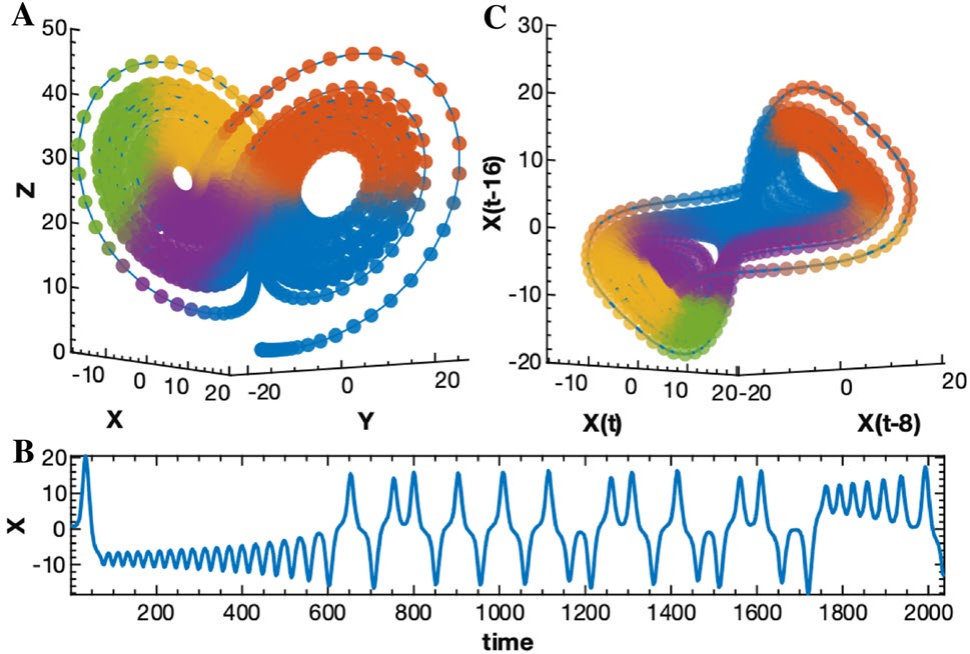
Illustration of Takens’ theorem. (**A**) A Lorenz attractor with time points colored according to proximity. (**B**) Time series of the $x$ coordinate. (**C**) Delay reconstruction from lagged $x$ coordinates. The timepoints have the same colors as those in (**A**). Notice how the delay reconstruction preserves the relative locations of the time points despite being transformed and warped. This demonstrates the homeomorphism between the two manifolds.

All SSR methods leverage Takens’ theorem to reconstruct the higher dimensional attractor of the dynamical system which generated an observed time series. This attractor is the manifold of points, $M$, visited by the system as it evolves through state space (Fig. 6A). State space is a Euclidean space with axes corresponding to the state variables, $\left\{x, y, z\dots \right\}$, of the system. Takens’ theorem shows that one can produce a topology preserving embedding of $M$ using delayed values of just one of its variables as surrogate coordinates. This means that there is a homeomorphism, or a smooth, invertible mapping, between the system’s trajectory in the coordinates $\left\{x, y, z\dots \right\}$ and $\left\{x\left(t\right), x\left(t+\tau \right), x\left(t+2\tau \right),\dots \right\}$ (Fig. 6B, C)^29^. Furthermore, since homeomorphisms are transitive, the reconstructions created from each variable will all be homeomorphic to one another. SSR methods rely on this transitivity by testing if there is a smooth mapping, $\sim$, between the manifolds, ${M}_{x}$ and ${M}_{y}$, reconstructed from two different variables, $x$ and $y$. If ${M}_{x}\sim {M}_{y}$, then $x$ and $y$ are likely components of the same dynamical system. In the case of unidirectional coupling, the driving variable is a component of the driven dynamical system but not vice versa. Therefore, there will only be a mapping from the driven variable to the undriven one^1,30^.

The primary challenge in determining if there is a smooth mapping between reconstructed manifolds is that their topologies are unknown. SSRs represent geometric point clouds whose topologies must be inferred from the distances between their points. It follows that a test for a smooth mapping must measure the correspondence between the relative location of time points in each reconstruction. That is, if time points are relatively close (as compared to all other points) in one reconstruction then, if there is a smooth map, they should also be close in the other. Complicating this process is that many real-world variables that could benefit from SSRs tend to be noisy and sparsely sampled, which makes any resultant reconstruction only weakly representative of the true topology of the attractor.

**Figure 7:**
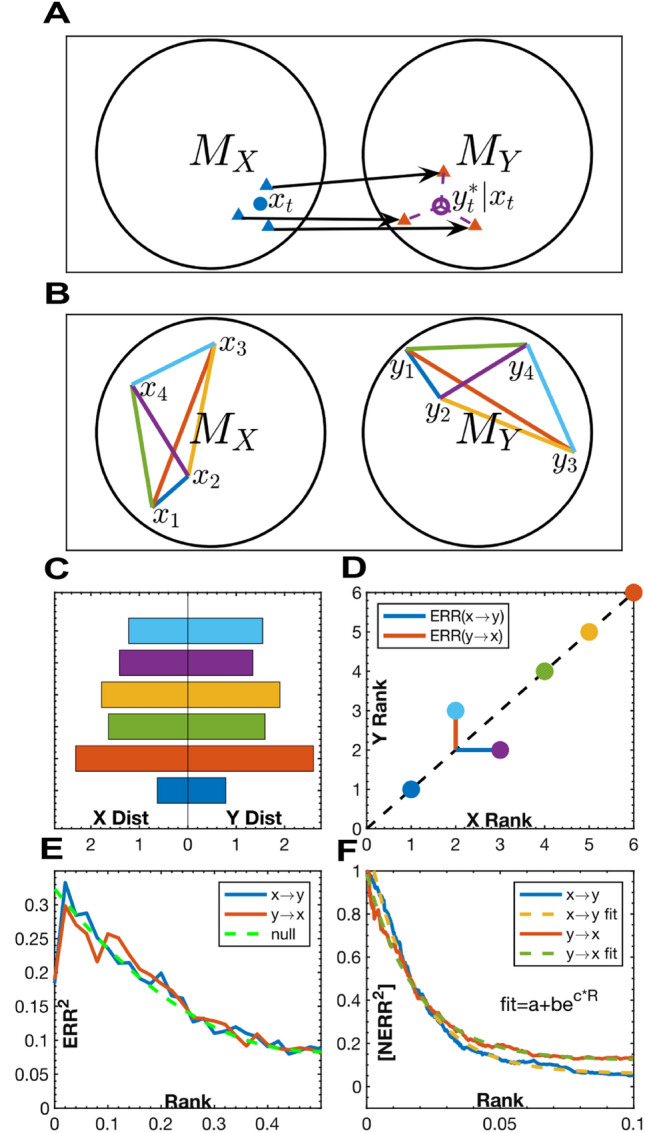
(**A**) An illustration of the CCM method. ${x}_{t}$ is point in ${M}_{X}$. The blue triangular markers represent its $D+1$ nearest neighbors where $D$ is the embedding dimension. The arrows show the mapping of each neighbor in ${M}_{X}$ to its location in ${M}_{Y}$. ${{y}_{{\varvec{t}}}}^{\boldsymbol{*}}|{x}_{t}$ is the estimate of the point ${y}_{t}$ from exponentially weighted average cross mapped neighbors from ${x}_{t}$. $CCM\left(y\to x\right)=corr\left({{y}_{{\varvec{t}}}}^{\boldsymbol{*}},{y}_{t}\right)$ (**B–F**) An illustration of the CCS method. (**B**) Pairwise distances between the same four timepoints in ${M}_{X}$ and ${M}_{Y}$ colored according to which time points they span. (**C**) The magnitude of the distances in both manifolds. (**D**) The rank of each distance in ${M}_{X}$, ${R}_{X}$, plotted against its rank in ${M}_{Y}$,${R}_{Y}$. The black dashed line represents perfect correspondence. The blue and red lines show the error between ranks, $ERR=$ ${R}_{X}$-${R}_{Y}$, as a function of ${R}_{Y}$ and ${R}_{X}$, respectively. $ERR\left(x\to y\right)= ERR\left({R}_{Y}\right)$ and $ERR\left(y\to x\right)= ERR\left({R}_{X}\right)$ because they measure how well ranks in the manifold of the driven variable predict ranks in the manifold of the driver. (**E**) ${ERR}^{2}$ as a function of rank for a bidirectionally coupled logistic map. The ranks have been normalized between 0 and 1. The dashed green line represents the null expected ${ERR}^{2}$ for uncorrelated ranks. (**F**) The cumulative average of the normalized error, $\left[N{ERR}^{2}\right],$ as a function of rank, for the system shown in (**E**). ${NERR}^{2}=(null-{ERR}^{2})/null$. $\left[N{ERR}^{2}\right]$ is thresholded at a maximum rank and fit to an exponential curve. The CCS scores are given by the y-intercepts of the fitted curves. Extrapolating from the best fit curve improves the estimate of the local correspondence by leveraging information from larger scales to overcome the high variance in ${NERR}^{2}$ observed at very low ranks.

CCM tests for this correspondence using the nearest neighbors (NN) of contemporary time points in each manifold (Fig. 7A)^1,8^. The NN implementation suffers from several practical problems. First, it requires a large number of points for the manifold to be densely sampled enough for the neighbors to be meaningful. Second, it fails to accurately estimate the interactions between strongly coupled or synchronous variables because their neighborhoods diverge at longer distances than those being considered^8,13^. This problem extends more generally to all oscillatory signals whose nearest neighbors tend to be points close in time. Finally, its estimates are degraded by noise, and are unreliable for stochastic systems^13^.

CCS overcomes these challenges by taking a more global perspective. Instead of just using local neighborhoods, it tests for a correspondence between the ranks of the pairwise distances between all the time points in each reconstruction (Fig. 7B–F, SI Convergent Cross Sorting). The primary advantage of this approach is that it creates a mechanism for only sampling the connections that are most informative of the topology of the space. By limiting the scope of the comparison to some lowest fraction of distances, CCS selectively considers the most densely covered portions of the manifold. This not only confers the direct benefit of eliminating the errors caused by outlying points but allows CCS to include far more pairwise distances than a KNN method because it doesn’t risk including more erroneous points. This means CCS can integrate more information from sparsely sampled manifolds which improves its performance on short and noisy data. It also enables CCS to test for the long-range divergences that are necessary for differentiating between strongly driven systems and bidirectional causation. Additionally, using ranks, instead of raw distances, normalizes for geometric transformations that distort absolute distance but preserve relative order. This has been shown to be a more reliable indicator of manifold structure^5^.

### Software

All analysis was performed using MATLAB R2020b available at https://www.mathworks.com/products/new_products/release2020b.html^31^. The MATLAB implementation of the CCS algorithm can be found at https://github.com/lbreston/CCS. Additionally, we used a vectorized version of the CCM algorithm available at www.mathworks.com/matlabcentral/fileexchange/52964-convergent-cross-mapping^32^.

### Ethics approval

All animal experiments were approved by the UCSD IRB. The experiments and maintenance procedures were performed in accordance with NIH and IACUC regulations. Surgeries were performed in accordance with UCSD IACUC animal welfare standards. Additionally, the study was carried out in compliance with the ARRIVE guidelines.

## Discussion

Non-linear dynamical systems analyses provide a vehicle for measuring coordinated activity across a variety of networks, whether they be social, behavioral, neural, or ecological. Rooting the analyses of these systems in SSR methods expands the possibilities for uncovering dynamic relationships, revealing structure that goes beyond coupling strength to address directionality. This paper compares the performance of the new CCS algorithm with CCM on simulated data from multiple model systems (VDP, LM and AR) where coupling parameters are known. In addition to the simulation results, we provide an exemplar application of CCS to systems neuroscience using an animal model informed by brain connectomics.

On the simulated data, CCS had higher accuracy than CCM for almost all test conditions. CCS saw its largest advantages on VDPs and short time series Logistic Maps. These results mean that CCS is particularly relevant to systems in which network states change very quickly, requiring high temporal resolution, and those that have smooth oscillatory components. CCS also retained much better accuracy on the stochastic ARs for which CCM fell below chance. Additionally, CCS was better at capturing the relative strength of bidirectional coupling for all but the lowest noise LMs. This may allow it to better capture the state of many real-world complex systems in which all the variables are coupled to some degree.

As an exemplar application to complex data, CCS was used to estimate bidirectional coupling between neural populations in the olfactory bulb, hippocampus and amygdala during social and self-grooming behavior. This application reinforced previous findings from the neurophysiology literature and provided further insight into the temporal dynamics of coupling strength and direction between brain regions during social interaction.

These regions switch rapidly from weak to strong oscillatory coupling. Whereas these oscillations typically maintain nested timescales, CCS was able to determine the strength and directionality of these interactions during social and self-grooming behavior. Grooming leads to a greater influence of autonomic input compared to social investigation, leading to stronger and more balanced coupling. These findings suggest that grooming is not only tactile and motor behavior, but also engages olfactory and autonomic processing.

The observed coupling dynamics changed across time and differed according to behavioral circumstances, where social behavior increased coupling lead by structures involved in affective appraisal and social memory. The pheromonal and olfactory stimuli encountered during social sniffing behaviors is highly salient compared to the other conditions tested, so this likely engages interaction with amygdala. The hippocampus also had increased influence on the olfactory bulb during social behavior as well, and this is likely associated with processes that underlie social memory formation. The results showed that there was increased influence in a primary sensory region from the amygdala, this may be due to the amygdala having a key role in saliency detection^33^. These findings further support the idea that amygdala, hippocampus and olfactory bulb are part of a memory network that elicits increased coupling in response to social stimuli^14,15^.

The experimental results demonstrate that CCS is a promising tool for uncovering dynamical relationships within systems that exhibit weak-to-strong coupling, rapidly changing network states, and/or oscillatory components. These systems include many types of physio-behavioral coupling both within and between individuals, and in larger groups or teams^34–36^.

CCS’s improved performance on dynamical and measurement noise, as well as coupled stochastic autoregression, suggest that it is useful for examining systems that are largely deterministic but contain some stochastic elements, such as commodity futures yoked to climate fluctuations.

CCS’s robust performance on a wide range of signals makes it a powerful tool for data analysis. It advances the state of the art by extending existing SSR methods to short, noisy, and oscillatory signals, greatly increasing the types of problems to which it applies. Furthermore, it is able to better distinguish relative coupling in bidirectionally coupled systems which improves its ability to reveal the coupling dynamics of real-world complex systems. We are releasing the method publicly such that other researchers can use CCS to investigate coupling within a diversity of nonlinear dynamical systems.

## Supplementary Information

Supplementary Information.
