# Supplementary material for: Convergent cross sorting for estimating dynamic coupling
Source: Sci Rep. 2021 Oct 13;11:20374. doi: 10.1038/s41598-021-98864-2 (PMC8514556; doi:10.1038/s41598-021-98864-2)
Supplement: Supplementary file 1 — Supplementary Information. [file 41598_2021_98864_MOESM1_ESM.pdf]

Supplementary Information for

Convergent Cross Sorting for Estimating Dynamic Coupling

Leo Breston, Eric Leonardis, Laleh Quinn, Michael Tolston, Janet Wiles, Andrea Chiba

Leo Breston
Email: lbreston@ucsd.edu

This PDF file includes:

Supplementary text
Figures S1-S4
Tables S1
SI References

Supplementary Information Text

Convergent Cross Sorting Algorithm

function CCS $(x, y, \ell, \mathcal{D}, \tau, T)$:

Input: x, y: Two timeseries of equal length

ℓ: Number of timesteps by which the timeseries are offset

$\mathcal{D}$: Embedding dimension

τ: Embedding delay

T: Fraction of pairwise distances considered

Output: $score(x \to y)$, $score(y \to x)$: CCS estimate of the coupling from $x \to y$ and $y \to x$

If T is not specified it must be chosen based on the roughness, ρ, of the signals. Rougher signals require a larger fraction of pairwise distances to be considered because their ranks are less tightly coupled.

1. $\rho = \max\left[\frac{\sigma(x')}{\sigma(x)}, \frac{\sigma(y')}{\sigma(y)}\right]$, $x' = \frac{dx}{dt}$ and $y' = \frac{dy}{dt}$ and $\sigma = \mathrm{SD}$
2. if $\rho \leq 1$ then $T = .05$ else $T = .1$

Lag and Embed x and y to create the phase space reconstructions.

3. $X(t - \ell) = [x(t),\ x(t + \tau), \dots\ x(t + (\mathcal{D} - 1)\tau)]$; $Y(t + \ell) = [y(t),\ y(t + \tau), \dots\ y(t + (\mathcal{D} - 1)\tau)]$

Find the pairwise distances between all the points in X and Y.

4. $D_x(i, j) = \mathrm{dist}(X(i), X(j))$; $D_y(i, j) = \mathrm{dist}(Y(i), Y(j))$ for $1 \leq i, j \leq \mathrm{length}(x)$

Remove the trivial elements surrounding the central diagonal of each distance matrix. These distances, between temporally close points, must be removed because they tend to be small regardless of manifold topology.

5. $\overline{D_x} = \mathrm{Upper\ Triangular}(D_x, k)$ and $\overline{D_y} = \mathrm{Upper\ Triangular}(D_y, k)$ where k is the first diagonal for which $\sigma(D_x(k)) \geq \sigma(D_x)$ or $\sigma\left(D_y(k)\right) \geq \sigma(D_y)$

Sort the remaining distances in ascending order and record their linear indices.

6. I_x and I_y are the lists of indices such that $\overline{D_x}(I_x)$ and $\overline{D_y}(I_y)$ are sorted

Normalize the distances to values between $(0,1]$ according to their ranks. Converting the distances to ranks improves the robustness of the algorithm because they are invariant to many geometric transformations.

7. $R = \frac{[1:\mathrm{length}(I)]}{\mathrm{length}(I)}$
8. $R_x = R(I_x)$; $R_y = R(I_y)$

Reorder the normalized ranks from each variable using the sorted indices from the other.

9. $R_{x \to y} = R_x(I_y)$; $R_{y \to x} = R_y(I_x)$

To estimate the asymmetric divergence between the manifolds; find the square error between the true, and cross sorted ranks for the lowest T fraction of distances.

10. $ERR^2(R_{x \to y}) = \left(R - R_{x \to y}\right)^2$; $ERR^2(R_{y \to x}) = \left(R - R_{y \to x}\right)^2$ for $R \leq T$

Normalize ERR^2 by the expected error of randomly distributed ranks. This step is necessary to appropriately weight the error across different scales, and to bound the measure between [-1,1].

11. $Null(R) = \mathrm{E}[ERR^2(R)] = R^2 - R + \frac{1}{3}$ for uncorrelated ranks
12. $NERR^2(R_{x \to y}) = \frac{Null - ERR^2(R_{x \to y})}{Null}$; $NERR^2(R_{y \to x}) = \frac{Null - ERR^2(R_{y \to x})}{Null}$

Find the cumulative average of $NERR^2$, as a function of R, to create a smoother estimate for how the expected error changes with manifold scale.

13. $[NERR^2(R_{x\to y})](R)$= $\frac{\sum_0^R NERR^2(R_{x\to y})(r)}{\sum_0^R r}$; $[NERR^2(R_{y\to x})](R)$= $\frac{\sum_0^R NERR^2(R_{y\to x})(r)}{\sum_0^R r}$

Fit $[NERR^2]$ to an exponential function of the form $f(R) = a + be^{cR}$. An exponential function was used because it was empirically observed that $[NERR^2]$ falls off exponentially for coupled systems. Since $[NERR^2]$ is a cumulative average, the points are weighted by $\sqrt{n}$ where n is the number of elements in the sum. The CCS scores are then given by the y-intercepts of the fitted curves.

14. $\text{score}(x \to y) = f_{[NERR^2(R_{x\to y})]}(0)$; $\text{score}(y \to x) = f_{[NERR^2(R_{y\to x})]}(0)$

The y-intercept is used as the CCS score because it captures the behavior of the error function as the distance between points becomes minimal. This reflects the topological property of local neighborhoods, which is the region of interest for testing smooth transformations.

Data Sets

Simulated Data

Van der Pol Oscillators

$$\frac{d^2x_i}{dt^2} = \mu(1 - x_i{}^2)\frac{dx_i}{dt} - \sum_{j=0}^{n} \kappa_{ij}x_j + \epsilon$$

VDPs were simulated using a fixed time step of $dt = .1$. x_i was randomly initialized between [0 1], $\frac{dx_i}{dt}$ was randomly initialized between [-1,1]. The self-coupling parameters, κ_{ii}, were randomly initialized between [.25, .75]. For all trials other than those evaluating the effect of coupling strength $K = .15$. For all trials other than those evaluating the effect time series length $L = 400$. For all trials not evaluating the effect of noise, $\varepsilon = 0$ and $\epsilon = 0$. Trials which diverged were rejected.

Logistic Maps

$$x_i(t) = x_i(t-1) * \left(r - \sum_{j=0}^{n} \kappa_{ij}x_j(t-1)\right) + \epsilon$$

x_i was randomly initialized between [0 1], $r = 3.8$, $\kappa_{ii} = 3.8$. For all trials other than those evaluating the effect of coupling strength $K = .1$. For all trials other than those evaluating the effect of time series length $L = 400$. For all trials not evaluating the effect of noise, $\varepsilon = 0$ and $\epsilon = 0$. Trials which diverged were rejected.

Autoregressive Models

$$\mathrm{x_i(t)} = \sum_{\mathrm{j=0}}^{\mathrm{n}} \kappa_{\mathrm{ij}}\mathrm{x_j(t-1)} + \epsilon$$

$\mathrm{x_i}$ was randomly initialized between [-.2 .2] The self-coupling parameters, κ_{ii}, were randomly initialized between [-1, 1]. For all trials other than those evaluating the effect of coupling strength $\mathrm{K} = .5$. For all trials other than those evaluating the effect time series length $\mathrm{L} = 400$. For all trials not evaluating the effect of dynamical noise $\epsilon = .1$. For all trials not evaluating the effect of measurement noise $\varepsilon = .1$. Trials which diverged were rejected.

20 Variable Networks

For each type of system, 20 variable networks were randomly initialized with a uniform coupling strength and a sparsity of 5%. 10% of those connections were then modified to be bidirectional, to ensure that causal loops would be represented in the data set.

Behavioral Neurophysiology Data

Social Interaction Task
A total of 10 Sprague-Dawley rats served as subjects in this experiment. Eight Sprague-Dawley rats (n = 8) acting either as novel or familiar rats (based on prior exposure) were included in the habituation experiment. Two rats (n = 2) were surgically implanted with electrodes for electrophysiological recordings. Two rats were placed in separate Plexiglas enclosures, while an implanted rat on the outside was free to roam the field and sniff through the holes in the enclosures containing other rats. The implanted rats were presented with both a novel and a familiar rat that they had previously met. The implanted rat freely roamed the field and investigated either the novel or familiar rat. Each trial lasted 2 minutes and 30 seconds; at the end of each trial rats were removed from the field. Trials were counterbalanced to control for place preferences, so novel and familiar rats were presented on alternating sides of the field with each trial.

Surgical Procedure
Two rats underwent surgery for electrode implantation in order to record simultaneous local field potentials from multiple brain areas. Surgeries were performed in accordance with UCSD IACUC animal welfare guidelines. Each rat was treated with isoflurane anesthesia (4-5% induction, 1-2% maintenance) and placed in a stereotaxic apparatus (Kopf Instruments) to allow for accurate placement of stereotrodes. Three holes were made through the skull (at the coordinates listed below), and the visible dura mater was removed. Anchor screws were inserted at four sites around the skull to support the implant. Reference and ground screws were inserted through a hole drilled above the cerebellum. Prior to surgery, stereotrodes were fabricated by twisting pairs of 50μm tungsten wire (California Fine Wire) and inserting them through polyamide tubing for insulation. Stereotrodes were implanted at the following coordinates (relative to Bregma): main olfactory bulb (8.5AP, 1.5ML, -3.5 DV), basomedial amygdala (-2.12AP, ±4.0ML, -9.2DV), and the CA1/CA2 region of the hippocampus (-3.8AP, 3.8ML, 3.2DV) laterally.

Neural Recordings
Each stereotrode was connected to a Neuralynx 16-channel electrode interface board (EIB-16) and amplified using the Neuralynx Cheetah-32 (Neuralynx Technologies, Bozeman, MT). Amplifiers were integrated with the Cheetah data acquisition software. The sampling rate for the recorded local field potentials was 1010.10Hz. Video was recorded from a camera above the field at 30Hz. Neuroexplorer was used to export the Neuralynx LFP channels to Matlab as a time series (NeuroExplorer; Plexon, Inc., Dallas, TX, USA).

Behavioral Video Coding
Video was annotated for behavioral events using ChronoViz (Fouse, Weibel, Hutchins, Hollan, 2011). Social sniffing was defined as the rat nose-poking the holes in the enclosure to sniff the enclosed rat; sniffs of the enclosure without nose poking were not included in the analysis. Grooming was coded as behavior involving small ellipses around the nose and mouth, lateral strokes with one arm at a time, then bilateral strokes with both arms, as well as body licking. Baseline behaviors were extracted during periods before the experiment began, when the rat was freely roaming the open field and not engaging in noticeable grooming or sniffing behavior. Events from annotations were divided into one second epochs (See Behavioral Epochs in SI for more information).

Animals and Housing
Ten Sprague-Dawley rats (n = 10) were used in the social interaction experiment, including two implanted rats and eight different rats presented inside the enclosures (Harlan Laboratories). All experiments and maintenance procedures were performed in accordance with NIH and IACUC guidelines. Rats were acquired at 6 weeks old and housed in pairs. Cagemates were put together in an enriched environment for 30 minutes a day and were maintained on a 12-hour light/dark cycle. After receiving surgery, the implanted

rat was single-housed for the remainder of the experiment, but still taken out to play in the enriched environment with the former cagemate on the same schedule.

Enriched Environment and Open Field

The open field consisted of a circular wood arena (114 cm diameter) that had a wall around the perimeter of the arena (24 cm height). The enriched environment consisted of an open field that contained multiple toys for rodents and a plastic hut.

Enclosures for Social Interaction Experiment

Plexiglas was cut with small holes in a grid pattern for all exposed surfaces of the cube shaped enclosure (Ridout). A Plexiglas base was also cut to support the walls and roof of the enclosure. Enclosures have a mechanical door on the side that the experimenter can open or close to let the rat out into the field after the trials concluded.

Behavioral Epochs

Video annotations of social sniffing, grooming, and baseline events were exported into Matlab. Due to a limited number of novel and familiar events, they were collapsed into a single social sniffing category for comparison with grooming and baseline behaviors. Events were of varying length; behaviors less than a second long were excluded from the analysis. Each event was divided into either one or multiple 1 second epochs, depending on length, and were separated based on behavioral category.

Histology

Analysis and Statistics

Method Implementations

CCS

The Matlab implementation of the CCS algorithm can be found at
https://github.com/lbreston/CCS

CCM

We used a vectorized version of the Matlab implementation of CCM available at:
www.mathworks.com/matlabcentral/fileexchange/52964-convergent-cross-mapping
(1).

Granger Causality

We used a Matlab implementation of Granger Causality available at:
https://www.mathworks.com/matlabcentral/fileexchange/59390-granger_cause_1
(2)

Method Parameters

See **Table S1**.

Choice of Embedding Parameters

To determine the best embedding parameters, we computed the false nearest neighbors (FFN), across a range of dimensions, $\mathcal{D}$, and delays, $\boldsymbol{\tau}$. The appropriate set of parameters should balance relatively few dimensions with a low number of FNNs. Simultaneously sweeping over both dimension and delay produces a more reliable parameter estimate than independently finding each parameter (3).

Theoretical Validation

Area Under the Curve 3 Variable

For each condition, the area under the curve (AUC) was calculated using 200 trials of three variable networks. Each trial had two randomly chosen coupled edges subject to the constraint that it could not contain transitive causality. CCS, CCM, and GC were used to estimate the coupling strength for each of

the 6 non-self-connections. Then Matlab's Perfcurve function was used to compute the AUC from the 1200 total estimates and their true coupling state. The confidence intervals were estimated with 1000 bootstrapped samples.

Area Under the Curve 20 Variable
For each condition, the area under the curve was calculated using 4 trials of 20 variable networks. CCS, CCM, and GC were used to estimate the coupling strength for each of the 380 non-self-connections. Then Matlab's Perfcurve function was used to compute the AUC from the 1520 total estimates and their true coupling state as determined by whether or not they were connected in the directed graph. The confidence intervals were estimated with 1000 bootstrapped samples.

AUC 20 Variable Results
To determine their performance on more complex systems we applied them to 20 variable networks with sparse connectivity and causal loops. **(SI 20 Variable Networks)** These results **(Fig S2)** show that pairwise tests lose their robustness under conditions with too many dimensions or hidden nodes. This limits their utility as a test set to distinguish between the different methods. It also emphasizes that it is critical to use pairwise tests in conjunction with prior knowledge of the connectome of the system. (4)

Bidirectional Coupling
All results shown in **Fig 3** were based on 10 trials per coupling condition. **Fig 3A** shows the average value of these trials. The confidence intervals for the spearman correlations in **Fig 3B** were calculated using the fisher transformation. The significance values in **Fig 3C** were calculated using a two-sample t-test.

Comparison of Spearman and Pearson Correlation
Fig S3 shows the Pearson correlation between the difference in estimated, and the difference in actual coupling strengths between bidirectionally coupled variables for CCS and CCM. The results are broadly consistent with the those from the Spearman correlation. A notable difference is that the performance on Logistic Maps converges for long time series lengths using the Pearson Correlation, whereas CCM maintains its advantage with the Spearman Correlation.

Neural Recordings
The results shown in **Fig 4** were based on 468 baseline, 457 grooming, and 140 social sniffing epochs. The significance values for **Fig 4B** were calculated from 10^5 bootstrapped samples taken from a null distribution of CCS scores. The CCS scores were derived from 100 temporally shuffled LFPs epochs. Prior to applying PCA the CCS vectors were normalized. The data shown in **Fig 4D** and **Fig 4E** was also whitened. The significance values in **4F** were computed empirically using a null distribution (10000 distributions of shuffled labels). The given p-value is the percentage of distributions with an nth most frequent sequence with a frequency as, or more, extreme than the one observed.

Comparison of CCS and CCM for Neural Data
For the comparison of the CCM and CCS for the neural recordings, CCS showed a larger sensitivity to differentiating between the bidirectional coupling in the brain regions with highest coupling scores, the hippocampus and the amygdala. This increase in sensitivity demonstrates the value of CCS's increased performance on systems that exhibit strong coupling.

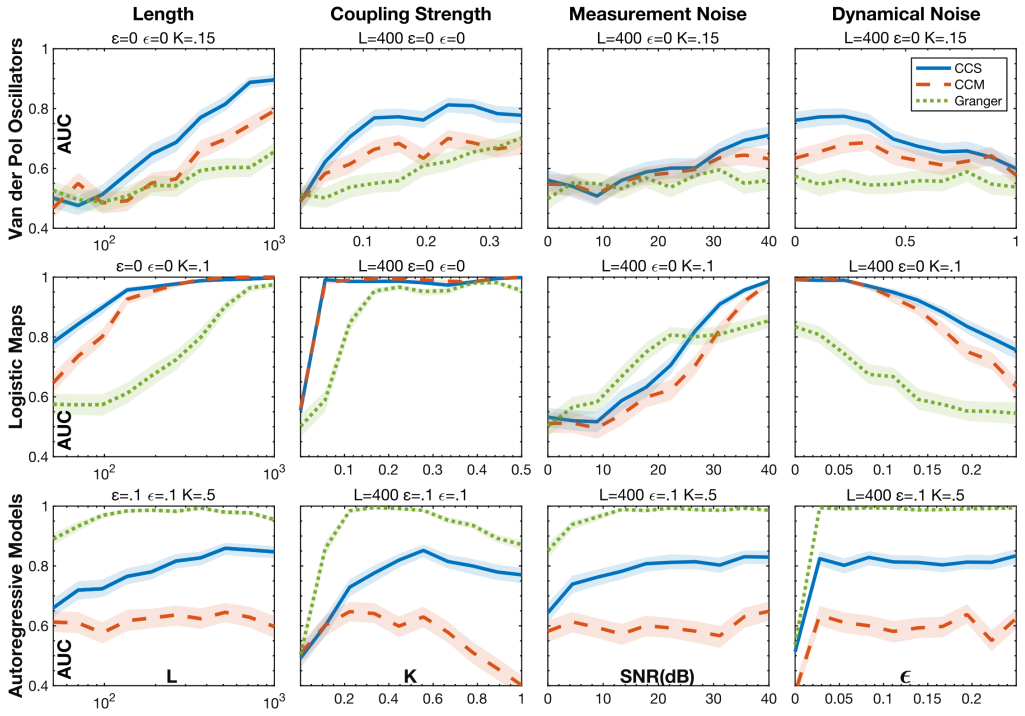

Fig. S1. Comparison of CCS and CCM to Granger Causality. The ROC AUC of CCS, CCM and GC for detecting causal coupling in three variable networks as a function of signal type, time series length, coupling strength, measurement noise, and dynamical noise. The shaded boundaries represent the 95% confidence intervals of the AUCs.

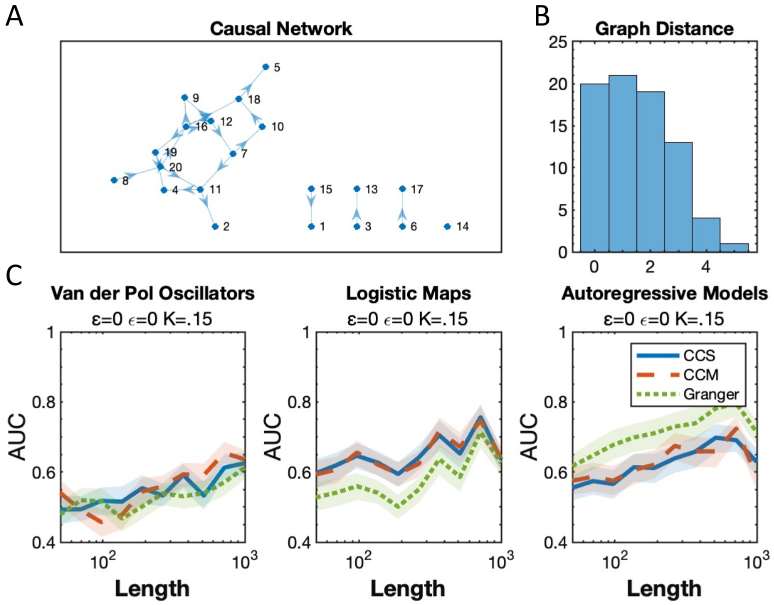

Fig. S2. Performance of different methods on 20 variable simulated data. **(A)** Example 20 causal network.(For network details see **SI 20 Variable Networks**) **(B)** Histogram of graph distance between variables in the causal network from **A**. This corresponds to the number of hidden nodes between coupled variables. **(C)** The ROC AUC of CCS, CCM and Granger for detecting causal coupling in 20 variable networks as a function of length. For each condition, the area under the curve (AUC) was calculated using 4 trials of 20 variable networks (See Supplementary **Area Under the Curve 20 Variable** for more information on the accuracy quantification and **Table S1** and **Choice of Embedding Parameters**). The shaded regions represent 95% confidence intervals.

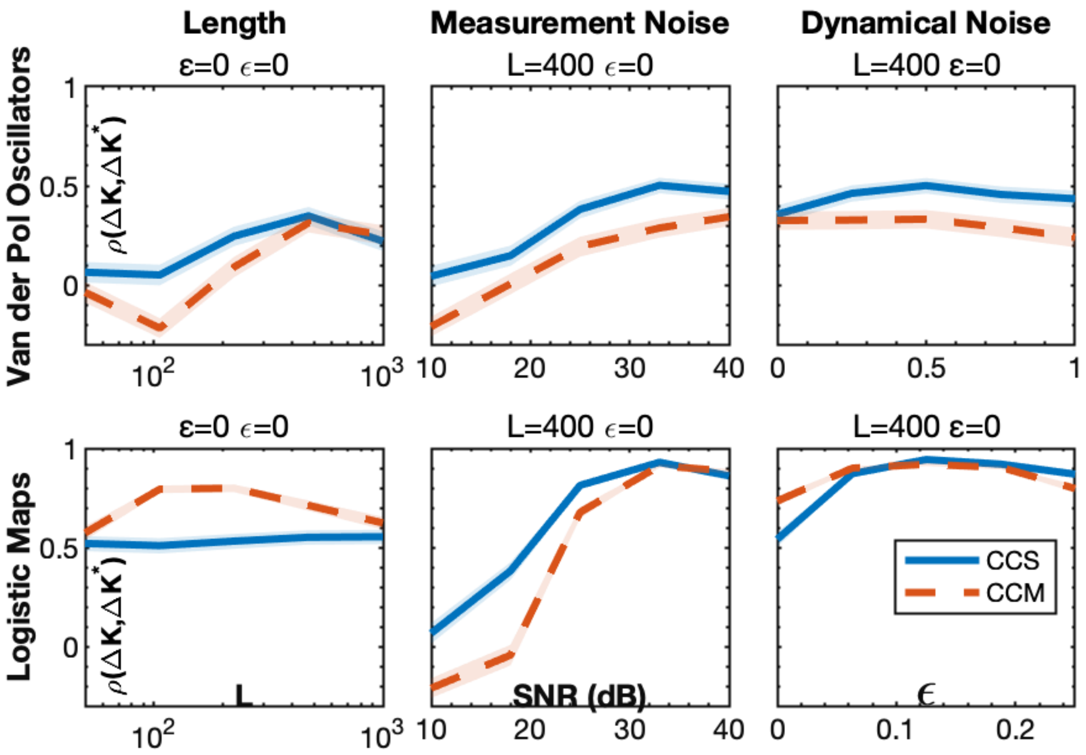

Fig. S3. The Pearson correlation between the true difference in coupling strength, $(K_{x\rightarrow y} - K_{y\rightarrow x})$ and the estimated one, $score(x \rightarrow y) - score(y \rightarrow x)$.

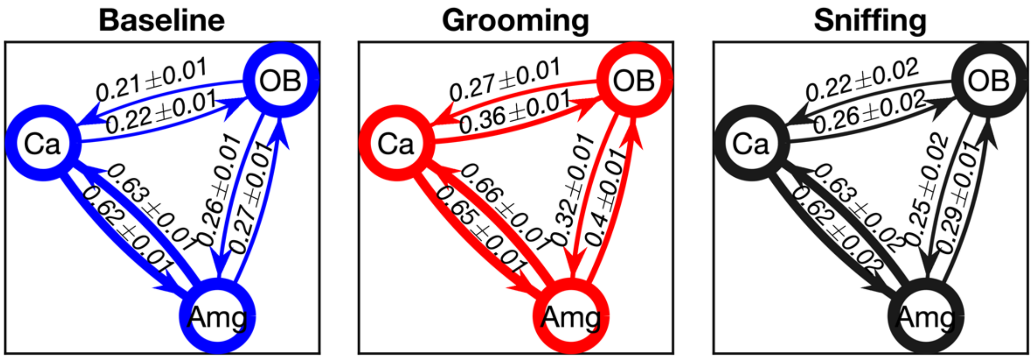

Fig. S4 Average 1 second CCM scores between the three regions during baseline, grooming, and sniffing behavioral epochs. (See Supplementary **Table S1** and **Choice of Embedding Parameters** for method details) Of note, CCM yields results similar to those of CCS. However, CCM underperforms in revealing the directionality of coupling in the most strongly coupled structures (amygdala and hippocampus).

Table S1.

Method parameters used for each data set.

	CCS	CCM	GC
VDP (3 variable)	Tau = 4, Dim = 4	Tau = 4, Dim = 4	Max lag = 10
LM (3 variable)	Tau = 1, Dim = 2	Tau = 1, Dim = 2	Max lag = 10
AR (3 variable)	Tau = 1, Dim = 5	Tau = 1, Dim = 5	Max lag = 10
VDP (20 variable)	Tau = 4, Dim = 4	Tau = 4, Dim = 4	Max lag = 10
LM (20 variable)	Tau = 1, Dim = 5	Tau = 1, Dim = 5	Max lag = 10
AR (20 variable)	Tau = 1, Dim = 5	Tau = 1, Dim = 5	Max lag = 10
Rat LFP	Tau = 10, Dim = 6	Tau = 10, Dim = 6	

References

1. Jakubik, J. (2020). Convergent Cross mapping (https://www.mathworks.com/matlabcentral/fileexchange/52964-convergent-cross-mapping), MATLAB Central File Exchange.
2. Robert (2021). Granger_Cause_1 (https://www.mathworks.com/matlabcentral/fileexchange/59390-granger_cause_1), MATLAB Central File Exchange. Retrieved July 20, 2021.
3. Krakovská, A., Mezeiová, K., & Budáčová, H. (2015). Use of false nearest neighbours for selecting variables and embedding parameters for state space reconstruction. Journal of Complex Systems, 2015.
4. Inferring causation from time series in Earth system sciences. J. Runge, S. Bathiany, E. Bollt, G. Camps-Valls, D. Coumou, E. Deyle, C. Glymour, M. Kretschmer, M.D. Mahecha, J. Munoz-Mari, E.H. van Ness, J. Peters, R. Quax, M. Reichstein, M. Scheffer, B. Schölkopf, P. Spirtes, G. Sugihara, J. Sun, K. Zhang, J. Zscheischler. Nature Communications 10: 2553 (2019).
